# Gram‐Scale Synthesis of Perdeuterated Paracetamol

**DOI:** 10.1002/jlcr.70034

**Published:** 2026-08-04

**Authors:** Alexander Park, Robert M. Edkins, June McCorquodale, James Tellam, Sarah Youngs, Katharina Edkins

**Affiliations:** ^1^ Strathclyde Institute of Pharmacy & Biomedical Sciences University of Strathclyde Glasgow UK; ^2^ Department of Pure & Applied Chemistry University of Strathclyde Glasgow UK; ^3^ ISIS Deuteration Facility STFC Rutherford Appleton Laboratory Didcot UK

## Abstract

The synthesis of deuterated compounds is a major stumbling block in the field of neutron total scattering on disordered materials. This is due to the requirement for large quantities of pure sample with high deuterium content across all positions. Typical deuterations in literature focus on site‐specific deuterations typically on milligram scales. Here, we successfully synthesised paracetamol‐*d*_9_, as well as its related isotopologues, on a multigram scale. We proceeded through an acid‐catalysed deuteration of 4‐aminophenol under microwave irradiation. We were able to efficiently purify the unstable intermediate, 4‐aminophenol‐*d*_4_ by sublimation, which meant we could avoid the formation of strongly coloured impurities that could not be removed from the final product otherwise. The pure intermediate then underwent acetylation with a 75% yield. More than 10 g of the final perdeuterated product was synthesised with an average deuterium incorporation of > 93% and no less than 78% at any individual position.

## Graphical abstract

We report the synthesis of perdeuterated paracetamol on a multigram scale with an average deuterium incorporation of > 93% and no less than 78% at any individual position.

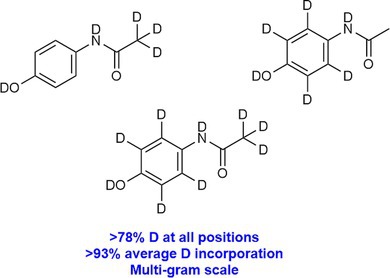

## 1 Introduction

Deuterium‐containing organic molecules are becoming increasingly common, with use as tracer molecules in pharmacokinetic studies [1], as substrates to investigate reaction kinetics [2], as standards for mass spectrometry [3] and more recently as active pharmaceutical compounds [4]. A less well‐known use of deuterated molecules is in neutron scattering experiments, where deuterated analogues are used to provide contrast in small‐angle neutron scattering experiments, to mask species in neutron spectroscopy or to gain more data on a single disordered system in neutron total scattering [5]. As neutrons interact with the nucleus of atoms within a sample, using different isotopes of the same atom, in this case protium and deuterium, will lead to different experimental measurables, which can allow for the separation of signal from different components in a mixture, such as the solvent and solute in a solution. In all neutron applications, deuterated samples are required to have high deuterium content at all hydrogen positions within the molecule, and often several grams of sample are required. Typical deuteration reactions are carried out on tens to hundreds of milligram scales and to achieve a high level of deuteration at all positions, harsh reaction conditions, platinum group catalysts and access to specialist equipment are necessary [6]. The harsh conditions can lead to the formation of impurities that are difficult to remove, making samples unsuitable for use in sensitive experiments. The need for catalysts and specialist equipment makes synthesis on a large scale costly and unviable for many.

Paracetamol, an antipyretic analgesic drug, is one of the most commonly used medications around the world [7]. It has regularly served as a model compound for many different studies involving neutron techniques [8, 9]. Despite this, there is little reported work on deuterating the compound. There are reports of synthesising the acetyl deuterated analogue through an acetylation of 4‐aminophenol (4‐AP) with acetic anhydride‐*d*_6_; however, no published work shows the synthesis of the perdeuterated drug.

In this work, we present a gram‐scale synthesis of paracetamol‐*d*_9_ with an average deuterium incorporation of > 93% and no less than 78% at any individual position. The route does not require the use of any transition metal catalysts and instead proceeds through an acid‐catalysed deuteration of 4‐AP under microwave irradiation, followed by an acetylation with acetic anhydride‐*d*_6_. Using this route, we also show the synthesis of the acetyl‐ and ring‐deuterated isotopologues of paracetamol.

## 2 Experimental

### 2.1 Materials

4‐Aminophenol, 1 M deuterium chloride in D_2_O and anhydrous sodium carbonate were purchased from Fisher Scientific; acetic anhydride*‐d*_6_, deuterated dimethyl sulfoxide (DMSO‐*d*_6_) and methanol‐*d*_1_ (MeOD) were purchased from Sigma‐Aldrich. Maleic acid was purchased from Fluorochem. All chemicals were used without any further purification.

### 2.2 Methods

#### 2.2.1 Calculation of Deuteration Levels

Measurements of deuterium levels were carried out by ^1^H NMR using a maleic acid internal standard. A known quantity of sample (approximately 40 mg) was weighed into a vial and added to a known quantity of maleic acid (approximately 20 mg). To that, 5 mL of DMSO‐*d*_6_ was added. A ^1^H NMR spectrum was acquired using a Bruker 400‐MHz spectrometer with BBO BBF‐H‐D iProbe using a single pulse to avoid the impact of T_1_ relaxation on the peak integrals. The signal at ~6.3 ppm corresponds to a two‐proton environment in maleic acid. The integral of this signal was compared to that from the sample, and differences in concentration and number of protons were accounted for.

#### 2.2.2 Deuteration of 4‐Aminophenol

4‐Aminophenol (1500 mg, 13.74 mmol) was added to a 20‐mL microwave vial along with a stir‐bar. The vial was sealed and purged with argon gas. Through the septum on the vial lid, 1 M DCl in D_2_O (13.74 mL) was added, and argon gas was bubbled through the solution. The vessel was placed in a Biotage Initiator+ synthetic microwave and reacted for 120 min at 175°C, reaching a maximum power of 175 W. During the reaction, the pressure increased to between 15 and 16 bar, and after 5 min, settled at 14 bar. After the reaction, the mixture was cooled to room temperature, and 1 M sodium carbonate solution was added dropwise. After the first additions, the reaction mixture changed to a cloudy pink colour. The addition was stopped once the pH of the mixture reached 8. The solid precipitate was collected and dried under vacuum, giving 4‐aminophenol‐*d*_4_ (4‐AP‐*d*_4_) as a pale pink to purple powder. The crude powder was purified by sublimation at 140°C at reduced pressure, yielding white dendritic crystals. For yields and deuteration levels of the first 10 reactions, see Table S1.

#### 2.2.3 Synthesis of Deuterated Paracetamol

In a round‐bottom flask under argon, 4‐aminophenol*‐d*_4_ (4.7512 g, 41.99 mmol) was dissolved in THF (60 mL). With stirring, acetic anhydride‐*d*_6_ (5.0101 g, 46.24 mmol, 1.1 mol equiv.) was added dropwise, ensuring the temperature did not rise significantly. After addition, the reaction was allowed to stir at 45°C for 90 min, after which the mixture was diluted with petroleum ether. A dark purple solid precipitate was collected and washed with petroleum ether (3 × 50 mL). TLC of the crude product, with acetone as the mobile phase, showed a single spot at *R*_f_ = 0.9 as well as a purple‐coloured spot on the baseline. The crude product was dissolved in acetone and vacuum‐filtrated through a 3‐cm‐thick layer of silica gel on a sinter funnel. The silica gel was then washed with acetone. A pale‐yellow solution was collected and reduced to dryness in vacuo, leaving an off‐white to brown crystalline product. These crystals were subsequently washed with ice‐cold water, giving pale pink crystals, which were then recrystallised from 1‐butanol, giving paracetamol‐*d*_7_ as a white powder (4.9327 g, 31.39 mmol, 74.75%). Paracetamol‐*d*_7_ was then dissolved in MeOD, stirred for 30 min, then the solvent was removed in vacuo. This process was repeated three times to give paracetamol‐*d*_9_ (92.3% yield).

This procedure was replicated using different isotopologues of 4‐AP and acetic anhydride: to give paracetamol‐*d*_5_ using 4‐AP and acetic anhydride‐*d*_6_, and paracetamol‐*d*_6_ using 4‐AP‐*d*_4_ and acetic anhydride.

## 3 Results and Discussion

**SCHEME 1:**
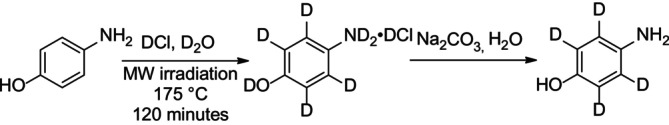
Deuteration of 4‐AP to 4‐AP‐*d*_7_ DCl, then work up with aqueous base to give 4‐AP‐*d*_4_.

Vaidyanathan et al. showed that 4‐AP could be successfully deuterated in DCl and D_2_O using microwave irradiation on a mmol scale [10]. Following a similar procedure, 4‐AP was dissolved in 1 M DCl in D_2_O and sealed in a microwave vial. The mixture was irradiated on medium power at 175°C in a microwave for varying amounts of time, resulting in different levels of deuterium incorporation (Table 1). After the reaction, 1 M Na_2_CO_3_ solution was added dropwise to the mixture until the mixture had a pH of 8. This caused the product to precipitate, and it was then collected and dried in vacuo (Scheme 1).

**TABLE 1:** Deuterium content of 4‐AP‐*d*_4_ with varied reaction times.

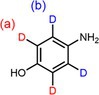		
Time (minutes)	Deuterium content (%)	
	Position a	Position b
20	16.44	92.51
2 × 20^a^	32.78	89.04
60	56.23	94.88
120	71.47	94.66
180	72.04	93.26

^a^ 20 min then worked up and rerun for 20 min with fresh DCl/D_2_O.

As mentioned previously, typical H/D exchange reactions require hydrogenation catalysts like palladium or platinum on carbon to facilitate the exchange. Due to the electron‐rich nature of the aromatic ring in 4‐AP, caused by the electron‐donating hydroxyl and amino groups, all the hydrogen positions are activated towards electrophilic aromatic substitution. This deuteration reaction was carried out with 1.5 g of reactant used at a time, and a reaction time of 2 h was found to give reasonable yields and deuteration levels (Table 2).

**TABLE 2:** Average yields and deuteration levels in 4‐AP‐*d4*.

Yield (%)	Deuterium content (%)	
	Position a	Position b
78.67 (±7.61)	73.22 (±2.92)	93.66 (±1.05)

**FIGURE 1:**
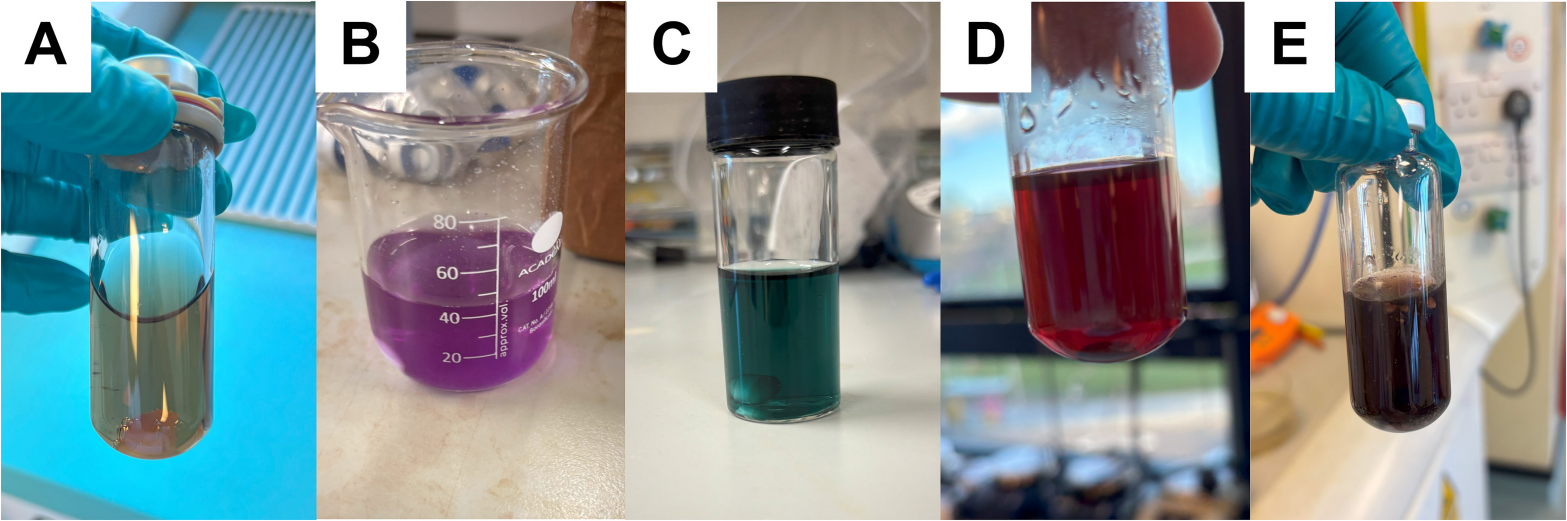
Results of the microwave‐assisted deuteration of 4‐AP (A) after 20‐min reaction with argon, (B–D) after 120‐min reaction under argon atmosphere and (E) after 120‐min reaction with air atmosphere.

Carrying out this deuteration left a black/deep purple solution (Figure 1E), which led to black crystals being isolated. Initially, it was thought that reacting the crude product with acetic anhydride to get paracetamol and then purifying that by crystallisation would be the most efficient pathway to gain a pure product, as its recrystallisation is well‐documented [11, 12, 13]. Upon carrying out the acetylation of 4‐AP with acetic anhydride‐*d*_6_, large dark crystals were formed. However, all attempts at recrystallising and washing the crystals failed, as the impurity seemed to incorporate itself into the crystal structure of paracetamol. The discolouration of the 4‐AP‐*d*_4_ most likely comes from oxidation; hence, the microwave vial and reaction mixture were instead purged with argon gas prior to the reaction.

The resulting solutions still discoloured; however, they took on a wide range of colours despite no apparent difference in reaction conditions (Figure 1). Using a reaction time of 20 min consistently gave a pale pink coloured solution, whereas using reaction times over 120 min gave solutions that were red, purple or dark green.

Upon slow addition of Na_2_CO_3_, the solutions that had been purged with argon quickly turned a cloudy pink colour, and the precipitate collected varied in colour from pale pink to pale purple. Addition of excess base, however, caused both the precipitate and the remaining solution to oxidise and turn dark purple.

It became apparent at this point that 4‐AP‐*d*_4_ degrades quicker than the protiated molecule. Samples left in solution turned black when left at room temperature for 4 h; similarly, the solid would turn black after 12 h when left exposed to an air atmosphere at room conditions. While 4‐AP is susceptible to degradation, it does not exhibit the same pronounced colour change after comparable periods of time. It is expected that the dramatic colour changes of the 4‐AP‐*d*_4_ are oxidation to the quinone‐imine, which may further polymerise, giving highly conjugated oligomers responsible for the intense deep purple or black colours formed. Why this reaction is facilitated in the deuterated over the protiated analogue is unknown, but it can be speculated that isotopic substitution on the hydroxyl and amino groups may slightly change the redox behaviour of the compound due to different bond energetics [2, 14].

Due to the inability to remove the remaining 4‐AP‐*d*_4_ impurities from the final perdeuterated paracetamol product, we decided to purify the 4‐AP‐*d*_4_ itself. Any attempt at recrystallising 4‐AP‐*d*_4_ did not improve the colour of the compound, often making it worse if it was left in solution for longer amounts of time. To avoid solution‐catalysed degradation, we used sublimation for purification. 4‐AP‐*d*_4_ sublimes readily, and initial tests showed that even at atmospheric pressure long dendritic crystals form with heating at 140°C, approximately 45°C below the melting point. This led to a huge loss of product as the majority of the sample degraded into a black solid at the bottom of the sublimation apparatus. Under reduced pressure, sublimation is far quicker and more efficient. At 90°C, up to 20 mg of purified product can be collected per hour. Increasing the temperature to 140°C increases the sublimation rate significantly. At 140°C, batches of 750 mg of 4‐AP‐*d*_4_ were placed at the bottom of a glass sublimation apparatus containing a cold finger, and after 1 h, 400 mg of white dendritic crystals were collected. Continuous collection over the next hour yielded a further 250 mg of product, with no further product subliming after this.

**SCHEME 2:**
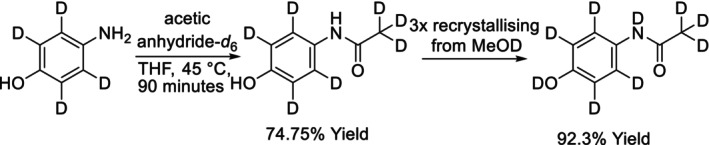
Synthesis of paracetamol‐*d*_9_ from 4‐AP‐*d*_4_ through acetylation with acetic anhydride‐*d*_6_ then recrystallisation three times from methanol‐OD.

**FIGURE 2:**
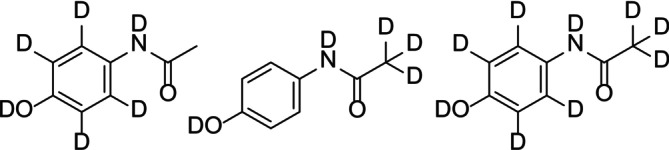
Isotopologues of paracetamol synthesised using different deuterated or protiated starting materials in the acetylation reaction.

The acetylation route shown in Scheme 2 allows access to different isotopologues of paracetamol to be synthesised (Figure 2) by using protium‐containing acetic anhydride or 4‐AP, which is of particular importance as contrasts in neutron scattering studies.

The purified 4‐AP‐*d*_4_ is reacted with acetic anhydride‐*d*_6_ in THF under argon atmosphere to give paracetamol‐*d*_7_. Despite using the pure white crystals of 4‐AP‐*d*_4_ and adding colourless acetic anhydride‐*d*_6_, the mixture quickly takes up a purple colour, which is retained. After 90 min, the mixture was diluted with petroleum ether, causing the product to precipitate as a light purple solid. In contrast to the paracetamol synthesised from crude 4‐AP‐*d*_4_, this discolouration can be purified by dissolving the crude product in acetone then filtering through a 3 cm layer of silica gel and subsequent drying in vacuo, which gives light brown crystals that turn pale pink upon washing with cold water. Recrystallisation from 1‐butanol then affords white crystals.

**FIGURE 3:**
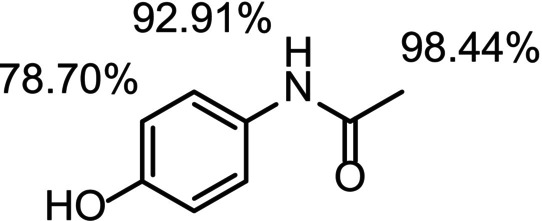
Final deuteration levels in paracetamol‐*d*_7_.

The final deuteration levels of the non‐exchangeable protons are shown in Figure 3. Deuteration levels *ortho* to the hydroxyl group (Figure 3) are notably higher than those determined for the 4‐AP‐*d*_4_ (Table S1). This is possibly due to the conversion of the 4‐AP‐*d*_4_ into a degradation product leading to different values being found (Figure S2). Further recrystallisation from MeOD was carried out three times to convert the exchangeable hydrogen positions to deuterium, giving paracetamol‐*d*_9_. The acetylation step was carried out on a 5 g scale with yields of up to 75%, allowing for the efficient synthesis of large quantities of pure sample required for neutron total‐scattering experiments.

## 4 Conclusion

Paracetamol‐*d*_9_ was produced on a gram scale through deuteration and purification of 4‐AP followed by acetylation with acetic anhydride‐*d*_6_. The final perdeuterated product had an average deuterium incorporation of > 93% and no less than 78% at any individual position. The 4‐AP‐*d*_4_ crude product was produced via a microwave reaction with deuterium chloride in D_2_O under argon to reduce oxidation byproducts. Furthermore, the crude 4‐AP‐*d*_4_ product was successfully purified by sublimation to give large pure white crystals, which could be a useful labelled precursor in the synthesis of biological ligands or small organic molecules that could be used in neutron scattering studies, as standards for mass spectrometry techniques or in drug‐metabolism studies. Without purification at this intermediate stage, a pure final product could not be achieved. The perdeuterated paracetamol and its related isotopologues themselves are important molecules for analytical chemists, which were previously only available to purchase on a milligram scale at high prices, which now can be synthesised at relatively low cost on a multigram scale.

## Author Contributions

**Alexander Park:** conceptualization (equal); investigation (equal); methodology (equal); writing – original draft (equal); writing – review and editing (equal). **Robert M. Edkins:** investigation (equal); methodology (equal); resources (equal); writing – review and editing (equal). **June McCorquodale:** investigation (equal); methodology (equal); resources (equal); writing – review and editing (equal). **James Tellam:** investigation (equal); methodology (equal); resources (equal); writing – review and editing (equal). **Sarah Youngs:** investigation (equal); methodology (equal); resources (equal); writing – review and editing (equal). **Katharina Edkins:** conceptualization (equal); investigation (equal); methodology (equal); resources (equal); funding acquisition; supervision; writing – original draft (equal); writing – review and editing (equal).

## Ethics Statement

The authors have nothing to report.

## Consent

All authors give consent for this work to be published.

## Conflicts of Interest

The authors declare no conflicts of interest.

## Supporting information


**Table S1:** Yields and deuteration levels of 4‐AP‐*d*_4_ produced.
**Figure S1:** ^1^H NMR spectrum of 4‐AP‐*d*_4_ in DMSO‐*d*_6_.
**Figure S2:** ^1^H NMR spectrum of 4‐AP‐*d*_4_ with and without maleic acid internal standard in DMSO‐*d*_6_.
**Figure S3:** ^1^H NMR spectrum of paracetamol‐*d*_9_ in DMSO‐*d*_6_.
**Figure S4:** IR spectrum of paracetamol‐*d*_9_.
**Table S2:** Microanalysis results of paracetamol‐*d*_9_.
**Figure S5:** ^1^H NMR spectrum of paracetamol‐*d*_7_.
**Figure S6:** IR spectrum of paracetamol‐*d*_7_.
**Figure S7:** ^1^H NMR spectrum of paracetamol‐*d*_*6*_ in DMSO‐*d*_6_.
**Figure S8:** IR spectrum of paracetamol‐*d*_6_.
**Figure S9:** ^1^H NMR spectrum of paracetamol‐*d*_*5*_ in DMSO‐*d*_6_.
**Figure S10:** IR spectrum of paracetamol‐*d*_5_.

## Data Availability

The data that support the findings of this study can be found at https://doi.org/10.15129/3944a4f1‐94df‐413c‐8759‐032a6bf41fab.
