# Supplementary material for: Gram‐Scale Synthesis of Perdeuterated Paracetamol
Source: J Labelled Comp Radiopharm. 2026 Aug 4;69(10-11):e70034. doi: 10.1002/jlcr.70034 (PMC13435339; doi:10.1002/jlcr.70034)
Supplement: Supplementary file 1 [file JLCR-69-0-s001.docx]

Gram-scale synthesis of perdeuterated paracetamol

Supplementary Information

Alexander Park^1^, Robert M. Edkins^2^, June McCorquodale^3^, James Tellam^3^, Sarah Youngs^3^, Katharina Edkins^1^

^1^ Strathclyde Institute of Pharmacy & Biomedical Sciences, University of Strathclyde, 161 Cathedral Street, Glasgow

^2^ Department of Pure & Applied Chemistry, University of Strathclyde, 295 Cathedral Street, Glasgow, G1 1XL, United Kingdom

^3^ ISIS Deuteration Facility, STFC Rutherford Appleton Laboratory, Harwell Oxford, Didcot

Contents

[Characterisation	2](#_Toc230333719)

[4-aminophenol-*d*_4_	2](#_Toc230333720)

[Paracetamol-*d*_9_	3](#_Toc230333721)

[Paracetamol-*d*_7_	3](#_Toc230333722)

[Paracetamol-*d*_6_	3](#_Toc230333723)

[Paracetamol-*d*_5_	3](#_Toc230333724)

| Table S1 – Yields and deuteration levels of 4-AP-*d*_4_ produced   | | | | | |
| --- | --- | --- | --- | --- | --- |
| **Batch** | **Starting material used (g)** | **Product (g)** | **Yield** | **Deuteration at position a** | **Deuteration at position b** |
| 1 | 1.5063 | 1.1252 | 74.70% | 71.5% | 94.7% |
| 2 | 1.5049 | 1.1669 | 77.54% | 72.0% | 93.3% |
| 3 | 1.5050 | 1.1244 | 74.71% | 70.3% | 93.8% |
| 4 | 1.5028 | 1.0854 | 72.23% | 70.4% | 91.4% |
| 5 | 1.5022 | 0.9476 | 63.08% | 75.9% | 93.0% |
| 6 | 1.5044 | 1.2525 | 83.26% | 70.4% | 93.5% |
| 7 | 1.5028 | 1.3101 | 87.18% | 77.8% | 93.5% |
| 8 | 1.5002 | 1.2486 | 83.23% | 75.6% | 93.9% |
| 9 | 1.5059 | 1.2798 | 84.99% | 71.6% | 94.3% |
| 10 | 1.4994 | 1.2859 | 85.76% | 76.6% | 95.3% |
| Average |  |  | 76.67% ± 7.22% | 73.2% ± 2.7% | 93.7 % ± 1.0% |

# Characterisation

^1^H NMR Spectrum

^1^H NMR spectra were acquired using a Bruker 400 MHz spectrometer with BBO BBF-H-D iProbe with 16 scans using a *zg30* pulse sequence. Spectra were acquired at 298 K.

IR Spectrum

Infrared spectra were acquired on a PerkinElmer Spectrum Two™ FTIR spectrometer. Spectra were measured within the range of 4000-400 cm^-1^ with 16 scans acquired at a resolution of 4 cm^-1^.

## 4-aminophenol-d_4_

Figure S1 – ^1^H NMR spectrum of 4-AP-*d*_4_ in DMSO-*d*_6_

Figure S2 – ^1^H NMR Spectrum of 4-AP-*d*_4_ with and without maleic acid internal standard in DMSO-*d*_6_

## Paracetamol-d_9_

Figure S3 – ^1^H NMR spectrum of paracetamol-*d*_9_ in DMSO-*d*_6_

Figure S4 – IR Spectrum of paracetamol-*d*_9_

Microanalysis

Elemental microanalysis was carried out using an Exeter CE-440 Elemental analyser. To get an accurate result, the value for hydrogen must be multiplied by a conversion factor of 1.9985 to account for the mass difference between protium and deuterium.

| Table S2 – Microanalysis results of paracetamol-*d*_9_ | | | |
| --- | --- | --- | --- |
|  | C | H/D | N |
| Theoretical H_9_ | 64.07 | 6.19 | 9.06 |
| Theoretical D_9_ | 59.97 | 11.31 | 8.74 |
| Results | 60.21 | 5.84 (11.67) | 8.64 |

## Paracetamol-d_7_

Figure S5 – ^1^H NMR spectrum of paracetamol-*d*_7_

Figure S6 – IR spectrum of paracetamol-*d*_7_

## Paracetamol-d_6_

Figure S7 – ^1^H NMR spectrum of paracetamol-*d_6_* in DMSO-*d*_6_

Figure S8 – IR spectrum of paracetamol-*d*_6_

## Paracetamol-d_5_

Figure S9 – ^1^H NMR spectrum of paracetamol-*d_5_* in DMSO-*d*_6_

Figure S10 – IR spectrum of paracetamol-*d_5_*
